# Clinical outcomes in cervical cancer patients treated by FDG-PET/CT-based 3-dimensional planning for the first brachytherapy session: Erratum

**DOI:** 10.1097/MD.0000000000016336

**Published:** 2019-06-28

In the article, “Clinical outcomes in cervical cancer patients treated by FDG-PET/CT-based 3-dimensional planning for the first brachytherapy session”,^[1]^ which appeared in Volume 95, Issue 25 of *Medicine*, Dr. Heerim Nam's affiliation appears incorrectly and should be Department of Radiation Oncology, Kangbuk Samsung Hospital, Sungkyunkwan University School of Medicine, Seoul, Korea.
